# Diagnostic utility of real-time RT-PCR for chikungunya virus detection in the acute phase of infection: a retrospective study

**DOI:** 10.1080/07853890.2025.2523559

**Published:** 2025-06-26

**Authors:** Anupama Sajith, Varsha Iyengar, Prasad Varamballi, Chiranjay Mukhopadhyay, Sudheesh Nittur

**Affiliations:** Manipal Academy of Higher Education, Manipal Institute of Virology, Manipal, India

**Keywords:** Chikungunya, arthralgia, laboratory diagnosis, real-time RT-PCR, AFI, good health and well-being

## Abstract

**Background:**

Chikungunya fever is a viral disease spread by Aedes mosquitoes, reported in over 110 countries. In India, it was first detected in Kolkata in 1963 and is now widespread. Diagnosis often relies on detecting anti-chikungunya IgM antibodies, but these may not be present during the acute phase as viremia can last up to 8 days, leading to underreporting. The present study aims to assess the diagnostic use of real-time RT-PCR for detecting chikungunya-specific nucleic acid in serum samples during the early stages of infection.

**Methodology:**

A retrospective cross-sectional study was conducted using archived samples collected as a part of ‘the hospital-based Acute Febrile Illness (AFI) surveillance project’. AFI cases having fever ≤8 days and arthralgia without any aetiology between 2016 and 2018 from Karnataka, Kerala, and Tamil Nadu were included in the study. Samples were subjected to nucleic acid extraction followed by chikungunya real-time RT-PCR using a standardized in-house protocol. The samples that tested positive for Chikungunya by real-time RT-PCR were further tested for detecting anti-Chikungunya IgM antibodies using enzyme-linked immunosorbent assay. Demographic characterization of the cases was performed using SPSS version 20 and GraphPad Prism version 10.

**Results:**

Out of a total of 646 samples tested, 31 samples (4.79%) were positive by real-time RT-PCR for chikungunya virus, 20 of which had a Ct value of <30, indicating a relatively high viral load. Among the 31 serum samples tested for anti-Chikungunya IgM antibodies, only one showed a positive result. Demographic analysis showed that 67% of cases were male and 32% were female, respectively. Clinical data analysis showed that most of the cases presented with cough (87%), headache (80.6%), myalgia (77.4%), coryza (70.9%), vomiting (58%), and abdominal pain (38.7%).

**Conclusion:**

The current study findings highlight the importance of screening patients with fever for up to 8 days and arthralgia for not only detecting IgM antibodies against chikungunya using ELISA but also for chikungunya virus-specific nucleic acid through real-time PCR/nucleic acid amplification techniques, or other methods. Not performing laboratory tests to screen for chikungunya-specific nucleic acid or antigen may result in underreporting of chikungunya cases, thereby impacting effective control measures and management of cases.

## 1. Introduction

Chikungunya fever is an arthropod-borne viral illness that has gained significant international attention since the early 2000s. This arboviral illness, which is spread by the Aedes mosquito, is characterized by symptoms like fever and joint pain. The name ‘chikungunya’ comes from the word ‘kungunyala’, which in the Kimakonde language means ‘to dry up or become contorted’. The disease’s characteristic arched posture is caused by rheumatologic symptoms known as ‘chikungunya’ [1]. The United Republic of Tanzania was the first nation to detect the virus in 1952, with more countries in Asia and Africa following. The first outbreak in urban areas was documented in India in the 1970s and in Thailand in 1967. There have been progressively constant and extensive chikungunya virus (CHIKV) outbreaks since 2004. This is mainly due to changes caused to the virus that increase the likelihood of infection transmission by *Aedes albopictus* mosquitoes. Currently, more than 110 nations in Asia, Africa, Europe, and the Americas have been found to harbour the chikungunya virus. India published the first chikungunya fever (CHIKF) reports in 1963. Following a 32-year hiatus, the chikungunya virus reemerged in India in 2005, leading to the most sig­nificant epidemic ever reported by 2006. The disease is currently prevalent throughout the nation, with outbreaks causing massive declines in production and the economy [2].

Over 160,000 cases of chikungunya disease and over 50 deaths have been documented globally as of March 31, 2024. The following nations have the most cases reported: Brazil (161794), Paraguay (5105), Bolivia (182), Argentina (272). In Asia, the most chikungunya cases were reported in India (154), Thailand (182), Pakistan (36), and Malaysia (13). Senegal was the only African nation that reported cases [3] of chikungunya infection in 2024 [4].

In India, according to the National Center for Vector Borne Diseases Control (NCVBDC), in 2018 Karnataka had the greatest number of laboratory-confirmed chikungunya cases (2546) followed by Gujarat (1290), Madhya Pradesh (1609) and Maharashtra (1009). In 2019, Karnataka had 3664 chikungunya confirmed cases, Maharashtra had 1646, and Telangana had 1358 confirmed cases. From 2020 to 2023, Karnataka had a higher number of confirmed cases (7736) than Gujarat (6664) and Maharashtra (6097), compared to other regions. Laboratory confirmation of chikungunya cases was done using chikungunya IgM MAC ELISA through the National Center for Vector Borne Diseases Control laboratory network [5,6].

The beginning of chikungunya virus disease in symptomatic patients usually occurs 4–8 days (with a range of 2–12 days) following an infected mosquito bite. It is characterized by a sudden onset of fever that is often accompanied by intense joint pain. Usually lasting a few days, the joint pain can be severe and continue for weeks, months, or even years at a time. The symptoms of chikungunya can vary from fever to complicated neurological conditions like encephalitis in rare cases [1]. Chikungunya disease is mostly found in regions where dengue fever is endemic and is transmitted by the same vector, the *Aedes* mosquito. Both occur in similar tropical and subtropical areas and present with similar symptoms. This overlap can sometimes result in misdiagnosis of chikungunya, as it may be mistaken for dengue fever. Considering the similar clinical characteristics of dengue and chikungunya, diagnostic testing is crucial, particularly in dengue-endemic areas [7]. Given the absence of specific antiviral treatments and the restricted availability of approved vaccines, especially for older adults due to safety concerns, it is necessary to emphasize the importance of accurate diagnostics to differentiate chikungunya from other febrile illnesses like dengue. Accurate diagnostics are pivotal for appropriate patient management and to prevent the misuse of treatments [8].

Several diagnostic tests are available for chikungunya, which include virus isolation, molecular assays and serological tests [9]. Among these methods, anti-Chikungunya IgM ELISA is the most commonly used diagnostic test [3]. Anti-chikungunya IgM antibodies typically begin to appear near the end of the first week of illness. Therefore, a convalescent-phase sample should be collected if the acute-phase sample tests negative to rule out the diagnosis. However, the viremia of chikungunya cases lasts up to 8 days, and it is difficult to detect anti-chikungunya-specific IgM antibody during the initial stages of infection. Numerous RT-PCR assays have been developed for detecting chikungunya virus, which are highly sensitive and specific. Therefore, detecting chikungunya-specific nucleic acid during the early phase of infection is imperative to estimate the extent of the current outbreak/epidemic and implement appropriate control measures [10,11]. In this study, we aimed to evaluate whether molecular diagnostics could be an useful tool during the acute phase of chikungunya virus infection.

## 2. Materials and methods

### 2.1. Selection of samples

**Figure 1.**
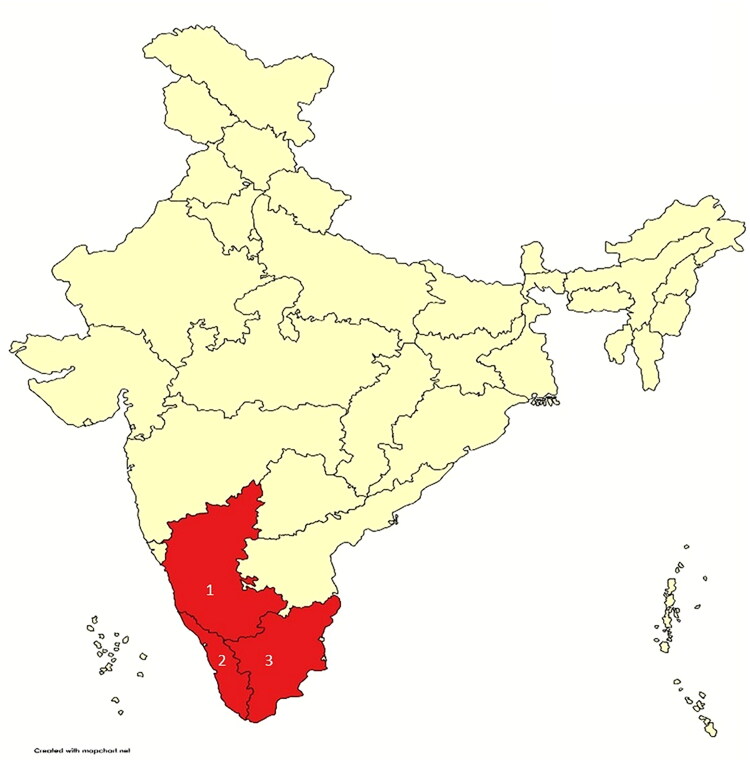
Geographical distribution of study sites highlighted in red (1. Karnataka, 2. Kerala, 3. Tamil Nadu).

A retrospective cross-sectional study was conducted using archived serum samples from Karnataka, Kerala, and Tamil Nadu collected as part of ‘the hospital-based Acute Febrile Illness (AFI) surveillance study between 2016 and 2018. Participants provided written consent, or for minors, their guardians provided consent through an assent form, which was collected during the hospital-based acute febrile illness study conducted between 2016 and 2018. The study was carried out following the principles outlined in the 2013 Helsinki Declaration [12] and after obtaining clearance from the Institutional Ethical Committee (IEC: 185/2024). All experiments were performed following relevant guidelines and regulations. A total of 689 serum samples were selected retrospectively and archived using the purposive sampling method, using the following inclusion criteria. This includes patients having fever ≤8 days and arthralgia without any etiology from South Indian states such as Karnataka, Kerala and Tamil Nadu from the period 2016 to 2018 (Figure 1) at the time of blood collection. Out of 689 serum samples selected, 43 were excluded due to insufficient volume. The remaining 646 samples collected during the acute stage of infection were retrieved from the biobank and used for the study.

### 2.2. Pooling of serum samples and nucleic acid extraction

All 646 serum samples were pooled by taking 5 samples in each pool, constituting 128 pools. The pooling strategy of the serum samples was performed following guidelines by ICMR [13]. Serum samples were pooled by mixing 50 microliters from each of 5 individual serum samples to create a pool with a total volume of 200 µL. From each pool, 140 µL was taken and subjected to nucleic acid extraction using a commercially available nucleic acid extraction kit (QIAamp Viral RNA nucleic acid extraction kit, catalogue number: 52906, Qiagen^™^, Germany) as per the manufacturer’s instructions.

### 2.3. Detection of chikungunya-specific nucleic acid using in-house chikungunya real-time RT-PCR targeting E1 gene (CHIK E1)

**Figure 2.**
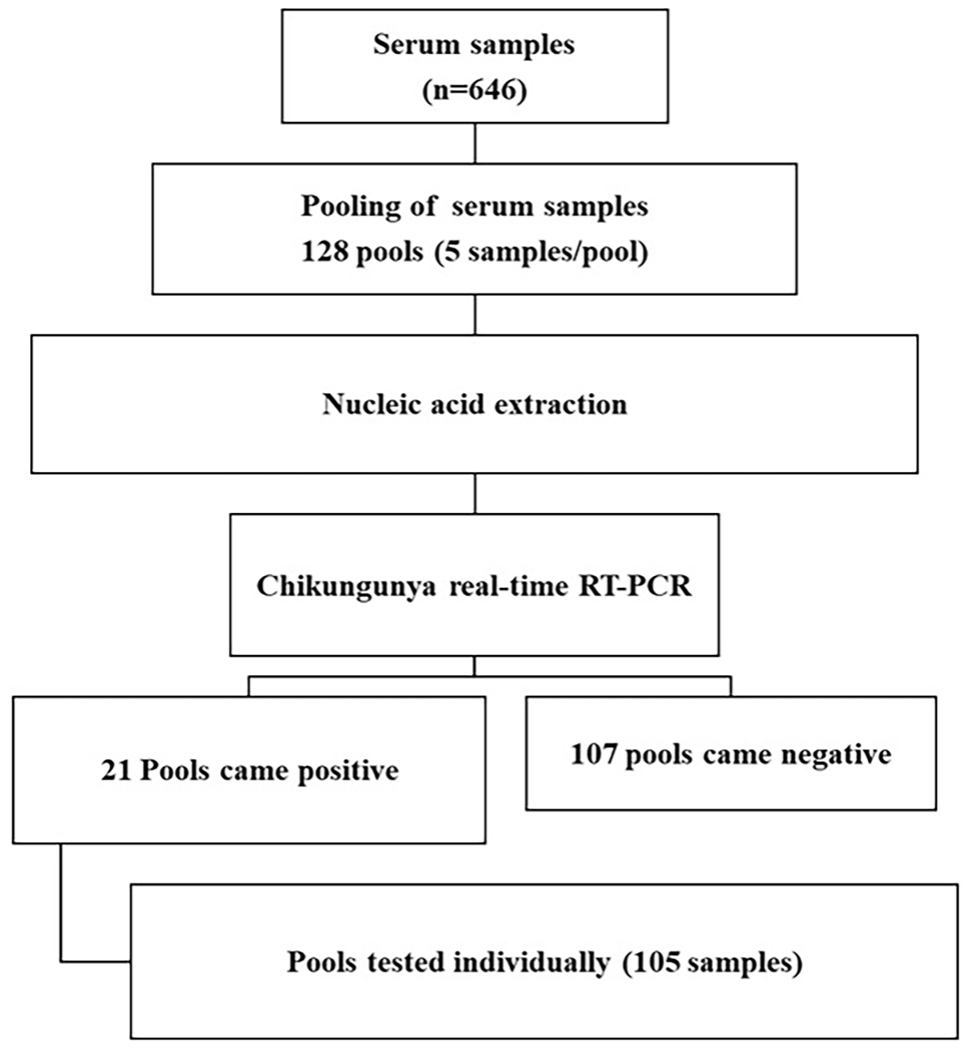
Workflow for nucleic acid extraction and real-time RT-PCR.

Extracted nucleic acid was subjected to chikungunya real-time RT-PCR, previously described by Edwards CJ et al. targeting the E1 gene [14]. The following in-house standardized protocol was used: a final reaction mixture with a total volume of 20 μL, which includes 0.2 μL of nuclease-free water, 11.5 μL of buffer mix (2X), 1.0 μL of each forward and reverse primers (10 µM), 0.5 μL of probe (10 µM), 0.8 μL enzyme mix (25X) and 5 µL of extracted nucleic acid. The AgPath-ID^™^ One-Step RT-PCR reagents (Applied Biosystems, USA) were used to perform the real-time RT-PCR. The cycling conditions used were 50 °C for 30 min, 95 °C for 15 min, 40 cycles of 95 °C for 15s and 58 °C for 30s. The reaction was performed in Applied Biosystems QuantStudio^™^ 6 Flex real-time PCR System using 6-FAM as reporter, NFQ-MGB as quencher and ROX as passive reference dye. The positive pools were selected, and individual samples from positive pools were further subjected to nucleic acid extrac­tion and real-time RT-PCR using the standardized protocol (Figure 2).

### 2.4. Detection of anti-chikungunya IgM antibody using ELISA

The real-time RT-PCR positive serum samples were subjected to anti-chikungunya IgM ELISA using CHIKjj Detect^TM^ IgM ELISA (catalogue no. DA6110, InBios International, Inc.,307 Westlake Ave N, Suite 300, Seattle, WA 98109 USA).

### 2.5. Demographic and clinical data analysis

Demographic data were collected and summarized using appropriate descriptive statistics: mean ± standard deviation (SD) for normally distributed continuous variables, median with interquartile range (IQR) for non-normally distributed continuous variables, and frequencies with percentages for categorical variables. Data analysis was performed using SPSS version 20. The chi-square test was employed to compare the distribution of clinical symptoms between chikungunya-positive and negative cases. Statistical analyses were conducted using GraphPad Prism version 10, and a *p*-value <0.05 was considered statistically significant.

## 3. Results

### 3.1. Detection of chikungunya-specific- nucleic acid using real-time RT-PCR

Out of 646 serum samples tested, 31 were positive by chikungunya real-time RT-PCR. All the positive samples had a Ct value ranging from 13 to 35. However, all 31 cases had a fever duration of less than five days (Table 1). Chikungunya infection was detected in 4.79% of patients by chikungunya real-time RT-PCR in South India (Karnataka, Kerala and Tamil Nadu).

**Table 1.** Demographic data and real-time RT-PCR Ct values of positive samples.

Sl. No	State	Duration of fever	Real-time RT-PCR results (Ct value)
1.	Tamil Nadu	4	30.4
2.	Tamil Nadu	3	13.8
3.	Tamil Nadu	5	34.4
4.	Tamil Nadu	3	17.5
5.	Tamil Nadu	4	28
6.	Tamil Nadu	3	15.4
7.	Tamil Nadu	2	17.7
8.	Tamil Nadu	5	22.6
9.	Tamil Nadu	3	26.2
10.	Tamil Nadu	3	19
11.	Tamil Nadu	2	16
12.	Tamil Nadu	4	34.5
13.	Tamil Nadu	3	28.2
14.	Tamil Nadu	3	28
15.	Tamil Nadu	3	28.7
16.	Tamil Nadu	3	29.59
17.	Tamil Nadu	5	35.2
18.	Tamil Nadu	4	34.5
19.	Tamil Nadu	3	26.5
20.	Tamil Nadu	3	27.9
21.	Tamil Nadu	2	22.2
22.	Tamil Nadu	3	26.5
23.	Tamil Nadu	3	31.1
24.	Tamil Nadu	3	19.5
25.	Kerala	3	34.2
26.	Kerala	3	33
27.	Kerala	4	34.5
28.	Kerala	5	33.2
29.	Karnataka	5	30.1
30.	Karnataka	3	15.2
31.	Karnataka	3	26.5

### 3.2. Detection of anti-chikungunya IgM antibody among real-time RT-PCR positive cases using ELISA

Only one out of 31 real-time RT-PCR positive serum samples tested positive for anti-chikungunya IgM antibody using ELISA. According to the kit interpretation, Immune status ratio (ISR) value ≥1 was considered as reactive (positive). Only one sample had an ISR value of 1.03, which had Ct value of 33.2 in real-time RT-PCR and had a history of fever for 5 days.

### 3.3. Clinical data analysis of real-time RT-PCR positive and negative cases

Among the 31 positive samples, all patients exhibited fever and arthralgia (100%), which was our inclusion criteria. Around 25 (80.6%) patients have shown headache, and 24 (77.4%) showed myalgia. Some patients were presented with abdominal pain (12/31, 38.7%) and vomiting (18/31, 58%). Few cases presented with neck stiffness (6/31,19.3%) and rashes in 2 (6.45%) cases. Apart from this, most of the cases presented with atypical clinical manifestations, like respiratory symptoms such as cough in 27 (87%) and coryza in 22 (70.9%) cases. Table 2 presents the distribution of clinical symptoms among chikungunya-positive (*n* = 31) and chikungunya-negative (*n* = 615) individuals (Table 2).

**Table 2.** Clinical data analysis of chikungunya real-time RT-PCR positive and negative samples.

Symptoms	No. of positive cases (*n* = 31) *N* (%)	No. of negative cases (*n* = 615) *N* (%)	*p*-Value
Fever	31 (100%)	615 (100%)	0.0368
Joint pain	31 (100%)	615 (100%)	0.9999
Cough	27 (87.1%)	547 (88.9%)	0.7679
Headache	25 (80.6%)	541 (88%)	0.2574
Myalgia	24 (77.4%)	527 (85.7%)	0.1979
Coryza	22 (71%)	364 (59.2%)	0.2598
Vomiting	**18 (58.1%)**	**186 (30.2%)**	**0.0023**
Abdominal pain	12 (38.7%)	172 (28%)	0.2213
Neck stiffness	6 (19.4%)	165 (26.8%)	0.4117
Rash (maculopapular rash)	**2 (6.5%)**	**5 (0.8%)**	**0.0403**

Statistically significant values were mentioned in bold.

Fever and joint pain were reported in 100% of cases in both groups; however, the difference in fever occurrence reached statistical significance (*p* = 0.0368), likely influenced by the large sample size of the negative group.

Vomiting was observed significantly more frequently among chikungunya-positive cases (58.1%) compared to negative cases (30.2%), with a *p*-value of 0.0023. Additionally, the presence of maculopapular rash was significantly higher in the positive group (6.5%) versus the negative group (0.8%), with a *p*-value of 0.0403.

Other symptoms, including cough, headache, myalgia, coryza, abdominal pain, and neck stiffness, did not differ significantly between the groups (*p* > 0.05).

These findings suggest that vomiting and the presence of maculopapular rash may serve as distinguishing clinical features in chikungunya-positive patients within the studied cohort.

### 3.4. Demographic data analysis of positive samples (*n* = 31)

The median age was 16 with an inter quartile range (IQR) of 10–30. Among these, 21 (67%) were male and remaining 10 (32%) were female. The cases were categorized into different age groups as per [15], aged from 5 to 9 years comprised of 16.12% (5/31), 10–14 were 22.5% (7/31), 15–19 were 22.5% (7/31), 20–24 were 6.45% (2/31), 25–29 were 9.6% (3/31), 30–34 were 6.45% (2/31) and remaining 5 cases (16.1%) were aged between 35 and 64s. All the reported cases were from the period 2017 and most of the cases were from Tamil Nadu. From total of 31 positives, 24 cases were from Denkanikottai (67%) and Anchetty (9.6%) regions of Krishnagiri district of Tamil Nadu state. Mananthavady region of Wayanad district, Kerala had 4 cases (12.9%) and Shivamogga district of Karnataka. which includes Soraba with 2 cases (6.4%) and Sagara with 1 (3.2%) case respectively.

## 4. Discussion

Chikungunya is an arboviral disease, which can cause severe joint pain and other symptoms. This can slowly lead to chronic joint pain and other neurological diseases like encephalitis in rare cases. Based on different studies conducted on chikungunya prevalence, studies have shown that the prevalence of chikungunya is high in Southern India [16]. The highest number of laboratory-confirmed cases was recorded in 2016, and this was followed by 2017 and 2019. The highest number of confirmed cases was reported from Karnataka, Maharashtra and Delhi. In 2019, a total of 12205 cases (14.9%) with laboratory confirmation of chikungunya was reported in 21 Indian states and 3 Union territories among 81914 patients who were clinically suspected of having chikungunya. Karnataka reported the highest number of cases of CHIKV (3664), with Maharashtra (1646), Telangana (1358), and Uttarakhand [1] reporting the lowest number of cases [17].

In our present study, we found that Southern India (Kerala, Karnataka and Tamil Nadu) has shown 4.79% of chikungunya infection among fever with arthralgia cases, and earlier studies conducted in 2012 also showed a RT-PCR and/or IgM-ELISA positivity with a rate of 49.3% [18]. There were reports of chikungunya outbreak in 2017 in Tamil Nadu and 2019–2020 in Kerala which showed PCR positivity rate of 90% which is followed by Karnataka, Telangana and Maharashtra with a PCR positivity rate of 15% [19]. In our study, we retrospectively analyzed the serum samples from a study conducted between 2016 and 2018, which included samples from Tamil Nadu, where an outbreak of chikungunya was reported in 2017, which correlates with the previous findings [11]. These findings indicate that Southern India continues to be endemic to chikungunya and has a high potential for future outbreaks, thus highlighting the importance of public health vigilance.

In our study, we noticed that most of the cases showed fever and arthralgia, which is the typical representation of chikungunya. However, our study also showed more respiratory symptoms of around 70–80%, which is a rare symptom of chikungunya. All these cases were negative for other respiratory viruses such as influenza virus, respiratory syncytial virus, rhinovirus, adenovirus, parainfluenza virus, coronavirus, human metapneumoviruses, as well as common respiratory bacterial pathogens. A case study reported in 2017 by Abhijeet has shown an unusual presentation of chikungunya having respiratory distress syndrome in a patient who was diagnosed with chikungunya fever [17]. Studies have shown that chikungunya can cause respiratory, renal, cardiovascular, neurological, ocular, neonatal infection with vertical transmission and skin manifestation [20]. While most people do not consider chikungunya to be life-threatening, atypical clinical manifestations can result in substantial morbidity and have been documented, particularly during epidemics [21]. Therefore, our findings indicate the importance of screening patients with respiratory symptoms with arthralgia for chikungunya viruses apart from common respiratory viruses. Here in our study, all the chikungunya real-time RT-PCR positive samples were negative for dengue NS1 antigen and anti-dengue IgM antibody by ELISA, as well as dengue-specific nucleic acid by real-time RT-PCR as most of the chikungunya affected areas overlap with dengue endemic regions, and this can cause the mosquito vector to carry both the agents. Moreover, they have indistinguishable symptoms and there may be chances of both the diseases getting misdiagnosed [7].

We also performed anti-chikungunya IgM antibody detection ELISA for real-time RT-PCR positive samples (*n* = 31), however, only one serum sample turned out to be positive with an index value just above the cut-off value. This sample had a Ct value of 33.2 in real-time RT-PCR with a history of fever for 5 days. These findings indicate the importance of screening for chikungunya-specific nucleic acid using real-time RT-PCR or other nucleic acid amplification techniques during the initial stage of infection, especially during the viremic phase. One limitation of our study is that we could not perform whole-genome sequencing of the positive cases due to limited funding. Moreover, the use of retrospective samples from 2016 to 2018 restricts our ability to conclude the current epidemiological status of chikungunya in India.

## 5. Conclusion

In conclusion, this study findings highlight the importance of screening patients with fever lasting up to 8 days and arthralgia for not only detecting IgM antibodies against chikungunya using ELISA but also for chikungunya virus-specific nucleic acid through real-time PCR/nucleic acid amplification techniques, or other methods capable of detecting chikungunya virus antigens. Such comprehensive screening is essential for accurately assessing the prevalence of the disease within a population, particularly in endemic regions, along with serological testing. Not performing molecular diagnostic/antigen detection techniques to screen for chikungunya-specific nucleic acid may result in the underreporting of chikungunya cases. Therefore, a com­­bined serological and molecular diagnostic approach can enhance case detection and provide a more accurate estimate of the disease burden.

Comprehending the prevalence of chikungunya enables effective utilization of resources, public health management and the development of targeted efforts by authorities, such as immunization campaigns or vector control strategies [22].

## Data Availability

The data and materials supporting the findings and analyses in this paper can be accessed upon request to the corresponding author, except where disclosure is restricted due to ethical, privacy or security concerns
